# Euthyroid Graves' Ophthalmopathy With Negative Antibodies (EGONA): A Comprehensive Case-Based Review

**DOI:** 10.7759/cureus.100152

**Published:** 2025-12-26

**Authors:** Maram Bonny, Mariam Wehbe, Rayan Zaydan, Mais Alsadi, Layan Hakim, Jana Abu Alhasan, Rashid S Abu Helwa

**Affiliations:** 1 Medicine, University of Sharjah, Sharjah, ARE; 2 Medicine, Emirates Health Services, Sharjah, ARE

**Keywords:** anti-thyroid peroxidase antibody, euthyroid graves' ophthalmopathy with negative antibodies (egona), grave's disease, graves' disease, graves' orbitopathy, thyroid stimulatory antibody

## Abstract

Euthyroid Graves' ophthalmopathy with negative antibodies (EGONA) is a rare form of thyroid eye disease. This literature review aims to establish the different presentations seen in the cases of euthyroid Graves' ophthalmopathy with negative antibodies. Despite the thyroid levels being normal, the mechanism by which EGONA causes eye disease is the same as the classical Graves' ophthalmopathy. Proper diagnosis relies mainly on clinical signs and imaging of the eye. A systematic search of PubMed, PubMed Central, and ScienceDirect was conducted with no date restrictions. Articles were included if they were English-language case reports describing patients with euthyroid ophthalmopathy and negative thyroid antibodies during initial presentation, with full-text availability and a documented clinical course. Data from nine articles (12 cases) were extracted, focusing on presentation, management, and outcomes. Treatments include corticosteroids, anti-thyroid medication, and surgery. Prognosis varied between those who developed thyroid imbalances and the presence of antibodies later down the course.

## Introduction and background

Definition and epidemiology

Graves' ophthalmopathy (GO) is the most common autoimmune eye condition [1]. It goes by many different names in the literature, such as Graves' orbitopathy, thyroid-associated ophthalmopathy, thyroid-associated orbitopathy, and thyroid eye disease, which all represent the same disease [2]. The key distinguishing factor among reported cases lies in the patient’s thyroid status at the time of ophthalmic presentation. The presentation varies into three different stages of being in a hyperthyroid, euthyroid, or hypothyroid state. Each was discovered to be prevalent in 86.2%, 7.9%, and 10.36%, respectively [3]. The incidence rate of this debilitating condition of the eye is 19 per 100,000 of the population [4]. GO is also the most common extrathyroidal manifestation of Graves' disease and is primarily characterized by autoimmune inflammation affecting orbital tissues. Although most cases occur in hyperthyroid patients, the disease can occasionally present in euthyroid or hypothyroid states, forming a small but clinically significant atypical subset.

Euthyroid Graves' ophthalmopathy (EGO) is a silent form of GO that can cause a similar eye disease seen in typical thyroid Graves' disease patients but without thyroid dysfunction [5]. In a study done in Singapore, they measured the thyroid status of 1020 patients with GO, and it was found that 0.7% of them presented with EGO [6]. EGO with Negative Antibody (EGONA) is a new term associated with the minimal yet very challenging presentation of thyroid orbitopathy in which no clinical explanation or investigation supports the diagnosis [7]. However, it becomes somewhat of a diagnosis of exclusion. This category specifically refers to patients who show clinical features highly suggestive of GO while maintaining persistently normal thyroid function tests and repeatedly negative thyroid-stimulating hormone receptor antibodies (TRAbs). Because these patients lack both biochemical and serological markers, the presentation often leads to diagnostic delay or misclassification. Multiple pathophysiological hypotheses have been proposed - including subtle immune dysregulation, limitations in current TRAb assays, or the potential involvement of alternative autoantibodies - yet no mechanism has been definitively established. Only a very few cases are present in the literature about EGONA and prognostic markers, and presentations have been infrequently taken into consideration. Therefore, this review aims to analyze and gather all available evidence on the reported cases of EGONA to help establish more presentable and comprehensive data on the clinical presentations, diagnostic investigations, treatments, management, and, most importantly, the prognostic evaluation of those patients. Given the rarity of this presentation and the inconsistency of published data, summarizing the existing evidence is crucial to improving diagnostic clarity and guiding clinical decision-making.

What is present in GO’s clinical features

The presentation of GO varies in many different cases and could range from very mild to extremely severe vision-endangering symptoms. The most common mild symptoms are periocular swelling, eyelid retraction, corneal irritation, chemosis, and extraocular muscle dysfunction [8]. Severe presentation of GO is seen in 5% of cases and can encompass the following symptoms: corneal ulceration, excessive proptosis, congestion of the eye, and optic neuropathy, which can lead to vision loss. All these presentations can extremely affect the quality of vision and life of a patient. In a study determining the clinical features of EGO through a comparison with untreated cases of GO, proptosis and extraocular enlargement were concluded as the most common symptoms to be present, with a higher incidence observed in males [9]. Although EGONA appears to share similar ophthalmic manifestations with classic GO, its natural history remains insufficiently understood due to limited documented cases.

It has been concluded that following up with patients with EGO was essential, as they could present with thyroid dysfunction shortly after their euthyroid presentation. Additionally, 8-25% of the patients presented with any degree of thyroid dysfunction around 15-45 months after the presentation of EGO [10]. Tamai et al. monitored seven patients with initially normal thyroid function and ophthalmopathy [11]. The follow-up duration ranged from six months to three years, during which thyroid abnormalities manifested in four out of the seven patients. However, in another study, the presentation of EGO was followed up for more than 10 years and has never shown any thyroid dysfunction [12]. Early data suggest that the risk of future thyroid dysfunction in EGONA is variable, but overall prognostic patterns remain unclear, highlighting the need for clearer epidemiological and clinical profiling.

What is present in GO’s diagnostic features

TRAbs are found in nearly every case of Graves' disease and are one of the main diagnostic features of GO [2]. The study done in Singapore found that 83% of their EGO patients had a positive diagnostic lab result of either polyethylene glycol-extracted thyroid-stimulating immunoglobulin (PEG-TSI) or human TSH-binding inhibitory immunoglobulin (hTBII) [6]. Both PEG-TSI and hTBII are part of the different methods to test for TRAbs [13]. Multiple studies have also supported the claim that GO severity correlates more with the antibody titer level in a patient [14,15]. Another study even correlated the importance of TRAbs as a prognostic predictor in treating the patient’s ophthalmopathy [16].

However, emerging reports describe a subset of patients who present with clear clinical features of GO despite having no detectable TRAbs [7,17]. The insufficient evidence and limited number of case presentations resulted in significant diagnostic uncertainty when treating and diagnosing patients with EGONA. Because these patients repeatedly test negative for TRAbs using current methodologies, clinicians must rely heavily on clinical evaluation, imaging findings, and exclusion of alternative causes of orbital inflammation. This diagnostic ambiguity underscores the need for standardized criteria tailored to EGONA. This case-based review specifically focuses on these antibody-negative euthyroid presentations, aiming to address the gap in current knowledge and improve diagnostic clarity for this atypical and diagnostically challenging subset of GO patients. By synthesizing available evidence, this review also aims to highlight the clinical implications of EGONA, identify patterns in reported outcomes, and propose considerations for future clinical practice.

## Review

Methodology

A literature review was conducted using PubMed, PubMed Central, and ScienceDirect to identify published case reports on EGONA. To ensure comprehensive coverage of terminology variability, particularly the inconsistent use of MeSH terms in this area, we used a broad search strategy combining keywords and alternative terms, such as “euthyroid Graves' ophthalmopathy”, “euthyroid orbitopathy”, “antibody-negative thyroid eye disease”, and “negative thyroid antibody”.

**Figure 1:**
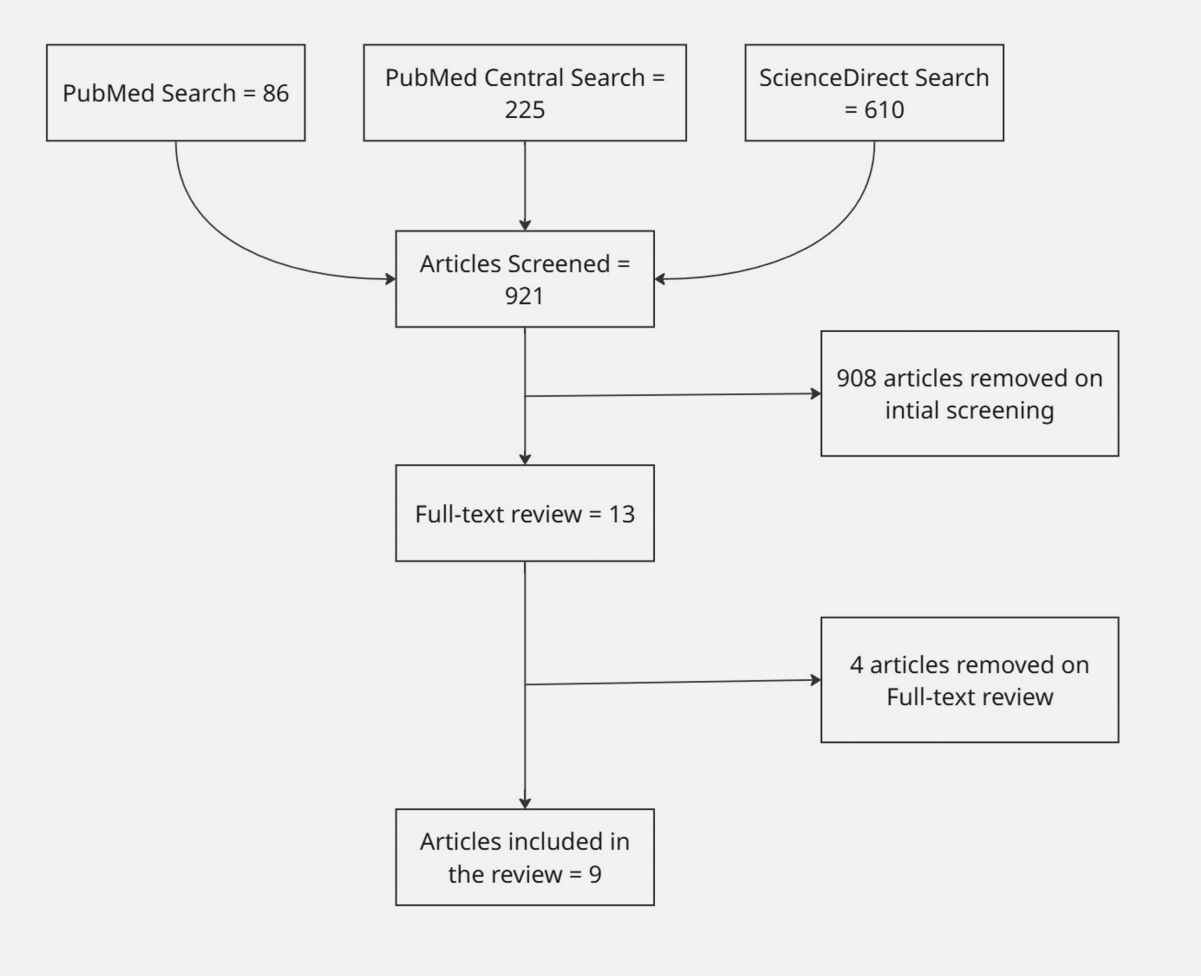
The figure represents the data collection process, including the used databases (PubMed, ScienceDirect, and PubMed Central) and the number of included and excluded articles

All retrieved articles were screened by title and abstract, followed by full-text review. Inclusion criteria consisted of English-language case reports describing patients who presented with ophthalmopathy while euthyroid and lacked detectable TRAbs at initial evaluation, with full-text availability and a documented disease course. Articles were excluded if they lacked confirmed euthyroid status, did not report thyroid antibody results, were not full-text accessible, were not in English, or did not provide sufficient clinical detail to evaluate the patient’s presentation and course. From the initial search (86 PubMed, 225 PubMed Central, 610 ScienceDirect results), nine articles meeting criteria were included, representing 12 distinct cases. All of this can be seen in the concept map in Figure 1.

Demographic

A total of 12 cases (six case reports and six case series) fit the criteria of EGONA, as presented in Table 1. A range of 24-84 years of age was present in the demographics of the patients. The average age was 46 years, and the median was 45. The cases consisted of six males and six females in total.

**Table 1:** Author, case number, year, and number of patients for the articles reviewed

Author	Case Number	Year	Patient #
Abu Helwa et al. [18]	Case 12	2024	1
Saleh Alfadl et al. [19]	Case 4, 5	2023	2
Cakir [17]	Case 8	2005	1
Margvelashvili et al. [20]	Case 3	2023	1
Mavridis et al. [21]	Case 9	2020	1
Moeen et al. [22]	Case 2	2021	1
Saha et al. [23]	Case 10, 11	2022	2
Santos et al. [24]	Case 6, 7	2019	2
Tabasum et al. [7]	Case 1	2016	1

Signs and symptoms

Signs

A review of 12 cases of EGONA from the literature revealed various signs and symptoms, highlighting the condition’s variability in presentation.

The most frequently observed sign was proptosis, reported in 11 cases (92%; cases 1-5 and 7-12). Among these, three cases (cases 2, 7, and 11) exhibited bilateral proptosis. Unilateral proptosis was more common in the right eye, occurring in six cases (cases 1, 5, 8, 9, 10, and 12), whereas left eye proptosis was observed in only one case (3). Case 5 was particularly notable for severe proptosis of the right eye, accompanied by early signs of proptosis in the left eye. In contrast, case 4 presented with mild proptosis, though the affected eye was not specified.

The second most frequently observed signs were lacrimation (excessive tearing) and eye erythema, each occurring in five cases (42%). Lacrimation was noted in cases 1, 2, 8, 9, and 10, while eye erythema was noted in cases 1, 2, 6, 9, and 12. The specific presentations of erythema varied, including erythema below the right inferior orbital rim (case 1), conjunctival erythema (case 2), periorbital erythema (case 6), bulbar conjunctival injection (case 9), and erythema of the right eye (case 12).

Following proptosis, lacrimation, and erythema, lid retraction was observed in two cases (17%; cases 9 and 10). It affected the right eye in case 10, whereas the affected eye was not specified in case 9. Lid lag was also present in two cases (17%; cases 8 and 9), with the right eye involved in case 8. Additionally, edema was observed in two cases (17%; cases 6 and 9), involving the left eye in case 6 and the eyelids in case 9.

Case 9 also exhibited abnormalities in eye movements, characterized by limitation of right eye abduction, along with a positive Ballet sign (restriction of one or more extraocular muscles) and a positive Gifford’s sign (difficulty in eversion of the upper lid). Furthermore, case 9 exhibited increased resistance and pain to retropulsion.

Less commonly observed signs included intracranial hypertension, dystopia, and optic atrophy, each seen in one case: intracranial hypertension (case 4), dystopia (case 5), and optic atrophy (case 2). All the symptoms are listed in Table 2.

**Table 2:** Observable signs in 12 reported cases

Signs	# Patients	% of the Total
Proptosis	11	92
Lacrimation	5	42
Eye erythema	5	42
Lid retraction	2	17
Lid lag	2	17
Edema	2	17
Abnormal movement	1	8
Resistant Retropulsion	1	8
Intracranial hypertension	1	8
Dystopia	1	8
Optic atrophy	1	8

Symptoms

The most commonly reported symptoms were diplopia and orbital pain, with orbital pain occurring in seven cases and diplopia in six. Diplopia was noted in cases 1, 3, 5, 9, 10, and 12, with case 10 specifying it affected the right eye, while the other cases did not specify. Orbital pain was recorded (cases 1, 2, 6, 8, 9, 10, and 12). Notably, two cases (cases 1 and 10) experienced pain in all directions of gaze, whereas, in case 12, there was right eye pain (radiating from a headache). Retrobulbar pain, periorbital pain, and right bulbar pain were each reported in one case (cases 2, 6, and 9, respectively).

The second most common symptoms were abnormal sensation in the eyes and impaired vision, each seen in 25% of the cases. Abnormal sensation was reported in cases 1, 8, and 10, with cases 1 and 10 describing it as a sticky feeling and case 8 describing it as a foreign body sensation. Impaired vision was noted in cases 2, 3, and 12. Case 2 reported bilateral vision loss and decreased visual acuity, while, in case 12, visual acuity was recorded as 20/150 in the right eye and 20/50 in the left eye, accompanied by photophobia.

The least common symptom was nausea, reported in only one case (case 12). Headache, on the other hand, was reported in three reports (cases 2, 4, and 12). Case 2 specifically mentioned an intermittent left-sided retroocular headache, whereas case 12 reported a four-day history of right temporal headache (10/10 in intensity) radiating to the right eye. Case 4 did not provide further details. All symptoms are listed in Table 3.

**Table 3:** Clinical symptoms in 12 reported cases

Symptoms	# Patients	% of the Total
Orbital pain	7	58
Diplopia	6	50
Abnormal sensation	3	25
Impaired vision	3	25
Headache	3	25
Nausea	1	8

Investigations

The investigations conducted after reviewing the 12 cases included thyroid hormone levels and other clinical tests. In terms of thyroid hormone levels, including T3, T4, and TSH, the following cases exhibited normal or lower levels despite presenting with exophthalmos (cases 1, 2, 8, 10, 11, and 12). For instance, the T4 values ranged between 0.90-1.34 ng/dL (cases 2, 10, 11, and 12) (reference range: 0.7-1.9 ng/dL) and 7.7 µg/dL (case 8) (reference range: 5.0-12.0 μg/dL). Meanwhile, the T3 values ranged between 2.75-3.5 pg/mol (cases 1, 10, and 11) (reference range: 2.3-4.1 pg/mL), and 95-138 ng/dL (cases 2 and 8) (reference range: 80-220 ng/dL). Moreover, the TSH hormone levels ranged between 0.855-2.25 µIU/mL (cases 8, 10, 11, and 12) (reference range: 0.27-4.2 uIU/mL), 2.25 U/L (case 1), and 1.98 mIU/L (case 2) (reference range: 0.5-5.0 mIU/L).

Other investigations that were carried out for case 10, such as calcium and ACE levels, were both within normal ranges (case 10). In addition, both TPOAb and TRAbs were within normal ranges in all cases, except in two particular cases. At first, both cases' TPOAb and TRAbs levels were normal at presentation; however, at follow-up and after treatment, these cases exhibited positive levels in cases 1 and 3, respectively. As for the TSAb levels, all cases exhibited normal levels. In case 1, a biopsy of the inferior rectus muscle showed a low-grade infiltrate with B and T lymphocytes (case 1). In case 12, an MRI scan was done, which showed fusiform thickening, with absence of masses, mostly appreciated on the left eye, in which the medial rectus with a 5.7 mm thickness (normal range: 3.7 ± 0.9 mm) and the inferior rectus with a 5.8 mm thickness (normal range: 4.0 ± 1.4 mm) were found to be the most thickened (case 12). Moreover, an ophthalmometer was used in case 2, and it measured the diameter of the eye to be 30 mm (normal range: 14-18 mm), which is higher than the normal range (case 2). Furthermore, an exophthalmometer was used in the following two cases (cases 6 and 11), and both of them exhibited exophthalmos of the right and left eyes measuring 15 mm and 19 mm, respectively (cases 6 and 11). The specific measurements are summarized in Table 4.

**Table 4:** Laboratory investigations of T3, T4, and TSH thyroid hormones Reference ranges: T3 (2.3-4.1 pg/mL) or (80-220 ng/dL). T4 (0.7-1.9 ng/dL) or (5.0-12.0 μg/dL). TSH (0.27-4.2 uIU/mL) or (0.5-5.0 mIU/L). Abbreviations: (TSH) Thyroid-Stimulating Hormone

Article	T3	T4	TSH
Tabasum et al. 2016 [7]	2.799 pg/mL	0.9012 ng/dL	2.25 U/L
Moeen et al. 2021 [22]	138 ng/dL	1.10 ng/dL	1.98 mIU/L
Cakir 2005 [17]	95 ng/dL	7.7 ug/dL	0.885 µIU/mL
Saha et al. 2022 [23]	2.8 pg/mL	1.28 ng/dL	2.25 µIU/mL
Saha et al. 2022 [23]	3.5 pg/mL	1.34 ng/dL	1.55 µIU/mL
Abu Helwa et al. 2024 [18]	—	1.24 ng/dL	1.25 µIU/mL

Recent updates on the investigations of EGO and EGONA

A retrospective cohort study introduced a new insight into diagnostic approaches toward the EGO. All patients included had a negative TBII, but with orbital symptoms, which do not exclude EGO. The available serum samples in the biobank were taken for the Turbo TSI bioassay, which detected TSAb in all samples, and, compared to the control group that had negative results, proved that there are no false positives. The Turbo TSI bioassay has a high sensitivity of 97.3%. Therefore, the Turbo TSI bioassay can provide earlier and more rapid results in diagnosing EGO compared to TBII or bridge-based TSI binding immunoassay [25].

Recent studies also suggest that orbital fibroblast activation plays an important role in the pathogenesis of EGONA, even in patients without detectable circulating thyroid antibodies. These fibroblasts can be activated by local inflammatory cytokines and immune cell infiltration, leading to increased glycosaminoglycan production, tissue expansion, and enlargement of the extraocular muscles, which are characteristic features of thyroid eye disease [26,27].

In addition, growing attention has been given to the insulin-like growth factor-1 receptor (IGF-1R) pathway. IGF-1R has been shown to interact with the thyroid-stimulating hormone receptor on orbital fibroblasts, enhancing inflammatory and fibrotic responses. Activation of this pathway may occur independently of traditional thyroid autoantibodies, which may help explain the development of ophthalmopathy in seronegative patients and supports the use of targeted therapies such as IGF-1R inhibitors, including teprotumumab [28,29].

Several potential biomarkers are also being explored to improve the diagnosis and monitoring of EGONA. These include serum IGF-1R autoantibodies and specific cytokine profiles that may reflect ongoing orbital inflammation. While these markers are not yet used in routine clinical practice, they may provide additional diagnostic value beyond conventional thyroid antibody testing in the future [30,31].

Radiological findings

In 10 out of the 12 collected case reports, the radiological findings are discussed in detail. The predominant modality used to check the changes in the recti muscles of the eye was MRI, in which 8 out of 10 cases used it to help assess the presence of changes. Five out of the 10 cases used CT. In three cases, they have used both modalities in their investigation.

Out of these 10 cases, only eight went into the details of the different sizes of each rectus muscle. The most commonly involved muscle, showing the greatest increase in size, was the inferior rectus muscle, which was affected in a total of six cases, either on its own by three reports (cases 1, 3, and 11) or joined by other muscles (cases 4, 9, and 12), which was always the medial rectus. Every other muscle was also involved at least once, as seen by the reported cases through both the CT and MRI modalities. No description was given comparing both modalities or emphasizing the importance of one over the other.

Finally, the eye that had the most prominent changes was the right eye, as it was involved in nine out of 10 cases that mentioned these changes. The left eye was involved in six of the cases. Both eyes were affected in five out of 10 cases in total.

We conclude that MRI was the modality of choice in most of the cases, although no data support the rationale behind this choice. Additionally, we deduce that the inferior rectus muscle is most frequently affected, with these changes predominantly occurring in the right eye.

Recent imaging in GO primarily serves as an additional means to the physician's assessment and clinical severity of the case, which still relies on standardized scores rather than on imaging‑based criteria. Current recommendations emphasize using orbital CT or MRI in atypical or unilateral cases, in euthyroid or antibody‑negative presentations where the diagnosis is uncertain, and whenever dysthyroid optic neuropathy or other sight‑threatening complications are suspected, as well as for pre‑operative planning before decompression. CT remains central for evaluating extraocular muscle enlargement, apical crowding, and bony anatomy, whereas MRI (especially fat‑suppressed T2 or STIR and contrast‑enhanced sequences) adds value by distinguishing active inflammatory changes from more fibrotic, inactive disease and by better characterizing soft tissues [32,33].​

Some work extends this role by exploring quantitative MRI (muscle volumetry, signal intensity metrics) and hybrid techniques such as FDG‑PET/MRI as research tools to refine assessment of inflammatory activity and to predict response to immunosuppressive or biologic therapy, although these methods have not yet been incorporated into formal scoring systems and remain largely investigational. Contemporary guideline comparisons show broad consensus that imaging should support, rather than replace, clinical staging and that its main contribution in challenging scenarios - such as euthyroid or seronegative GO - is to confirm the diagnosis, exclude alternative orbital pathology, and guide individualized management [34].

Management

The different proper measures taken in the case of management were divided into three main categories: radiation, surgical, and medical management. Eight cases out of the 12 reported details on their management approach. Out of the eight cases, seven of them went for medical management. Out of the seven cases, most have given steroids as a means of treatment. Four cases received oral steroids (cases 2, 6, 7, and 12), and three cases received intravenous steroid injections (cases 2, 3, and 11). Carbimazole was also given as the final treatment in two cases after initiating with other forms of treatment (cases 10 and 11).

Surgical treatment of the inferior rectus was done in case 1. It was also followed by the initiation of treatment with carbimazole after the hyperthyroidism symptoms had begun. Radiation treatment was documented by case 11, which was accompanied by pulse steroid therapy, as well as carbimazole being given at the end of the treatment.

No preferred outcome was established, but a clear number of cases favoured the use of steroid therapy as a major way of treating EGONA.

Prognosis

After reviewing 12 case reports, most did not go into details in terms of prognosis. Other cases, however, provided useful insight into the course of the disease. For example, some cases reported positive TRAb (cases 1, 3, 10, and 11), while case 12 reported negative TRAb, specifically after treatment. Regarding TPOAb, only two cases reported a positive finding upon follow-up (cases 1 and 3); the rest did not mention it. Cases 1, 10, and 11 reported low TSH levels and development of hyperthyroidism symptoms after 24 months, 8 months, and 10 months, respectively. In case 1, FT4/FT3 was reported normal/high (FT4: 15.5 pmol/L, FT3: 9.56 mol/L). In cases 10 and 11, on the other hand, it was reported that the levels were elevated (elevated FT4 or FT3 or both). Additionally, case 12 mentioned a negative TSI test, as the patient remained euthyroid. Finally, for ocular symptoms, it was only mentioned in two cases. Cases 2 and 12 reported that previous ocular symptoms improved, specifically by the third month for case 12.

Cases 6 and 7 only reported a good response to medication. Cases 4, 5, 8, and 9 offered no details regarding prognosis.

## Conclusions

EGONA is a rare and diagnostically challenging condition of thyroid eye disease, most consistently manifesting with proptosis and inferior rectus involvement. Among the 12 reported cases reviewed, some patients later developed thyroid abnormalities or subsequently tested positive for antibodies, which highlights the importance of long-term follow-up. Steroid therapy was the most common treatment, while surgical and radiation interventions were used less frequently.

This review is limited by the small number of available case reports and the lack of statistical data, which restricts generalizing these patterns and outcomes. Nevertheless, it contributes to the existing literature by bringing together what is known about the clinical features, diagnostic challenges, and disease course of this uncommon presentation. Overall, EGONA should be regarded as a diagnosis of exclusion requiring a high index of suspicion, multidisciplinary management, and careful monitoring. Further research, including larger studies as more cases are published, is needed to develop standardized diagnostic criteria and evidence-based management strategies for this unique subgroup of GO patients.
